# 以骨质破坏起病和嗜酸性粒细胞增多的PDGFRα重排髓系/淋系肿瘤1例

**DOI:** 10.3760/cma.j.cn121090-20231126-00279

**Published:** 2024-02

**Authors:** 燕 郭, 国庆 吕, 媛 张, 莉华 王, 金日 武, 秀利 卢, 卫玲 秦, 隼 吴

**Affiliations:** 1 新乡医学院第一附属医院血液科，卫辉 453100 Department of Hematology, the First Affiliated Hospital of Xinxiang Medical University, Weihui 453100, China; 2 新乡医学院第三附属医院血液科，新乡 453000 Department of Hematology, the Third Affiliated Hospital of Xinxiang Medical University, Xinxiang 453000, China; 3 新乡市白血病分子诊疗重点实验室，卫辉 453100 Key Laboratory of Molecular Diagnosis and Treatment for Leukemia in Xinxiang, Weihui 453100, China

患者，男，46岁，因“双髋部疼痛1个月”于2023年2月就诊于新乡医学院第三附属医院，完善双髋关节平扫+增强磁共振：右侧髂窝区、双侧髂骨、骶椎、双侧髋臼、右侧股骨头及小转子、双侧坐骨异常信号，疑诊为“多发性骨髓瘤”收入血液科。无皮肤瘙痒、咳嗽、胸闷、气喘、腹痛、腹泻等症状。既往体健；无药物过敏史；无吸烟、饮酒嗜好；无外地旅居史，无不洁饮食，未曾食用生冷海鲜。

查体：美国东部肿瘤协作组（ECOG）评分1分，一般状态差，贫血貌，全身浅表淋巴结未触及肿大，胸骨压痛阴性，双肺底呼吸音弱，心脏查体未见异常，腹部膨隆，肝脾肋缘下未触及，移动性浊音阳性，双下肢轻度指凹性水肿。入院后完善相关检查，血常规：WBC 37.45×10^9^/L，嗜酸性粒细胞42.1％（15.76×10^9^/L），HGB 97 g/L，PLT 120×10^9^/L；超敏C反应蛋白35 mg/L；肝功能：ALT 139 U/L，AST 68 U/L，白蛋白26 g/L，球蛋白38 g/L，碱性磷酸酶356 U/L；乳酸脱氢酶280 U/L；红细胞沉降率：65 mm/1 h；铁蛋白>3 000 ng/ml；心肌损伤标志物及B型脑钠肽（BNP）未见明显异常；呼吸道病毒、巨细胞病毒（CMV）、EB病毒（EBV）、寄生虫、降钙素原（PCT）、G实验、GM实验等感染相关检验均阴性；自身抗体谱18项、血管炎四项、肿瘤标志物、M蛋白筛查均阴性，血清游离轻链无限制性表达；骨髓象：有核细胞增生极度活跃，粒∶红比值为9.65，粒系占88.8％，红系占9.2％，形态均未见明显异常，其中嗜酸性粒细胞占24％，嗜酸性粒细胞颗粒、非特异性颗粒、中性颗粒溢出弥漫分布于全片；红系比例减低，以中、晚幼红细胞增生为主。外周血片：嗜酸性粒细胞比例增高，嗜酸性粒细胞杆状核占13％，分叶核占34％，胞质内可见空泡。骨髓活检病理：粒系增生伴嗜酸性粒细胞增多，红系、巨核系少见。骨髓流式细胞术免疫分型：可见表达紊乱的CD11b、CD13、CD16、CD15嗜酸性粒细胞占15.7％。BCR::ABL1融合基因阴性。荧光原位杂交（FISH）检测：PDGFRα基因重排占65％，FGFR1、PDGFRβ及JAK2基因重排阴性。骨髓染色体核型46, XY[20]。RT-PCR及直接测序证实CHIC2基因缺失及FIP1L1::PDGFRα融合基因阳性。胃肠镜检查提示：非萎缩性胃炎。CT引导下右髂骨穿刺物病理活检示大部分为出血伴纤维素渗出，小团纤维组织伴炎性细胞浸润。诊断为原发性高嗜酸性粒细胞增多症，给予伊马替尼100 mg每日1次口服治疗1个月，骨痛无明显改善，遂转院就诊于新乡医学院第一附属医院。入院后完善血常规提示：WBC 3.74×10^9^/L，嗜酸性粒细胞0.5％（0.02×10^9^/L），HGB 98 g/L，PLT 256×10^9^/L；肝功能：ALT 103 U/L，AST 51 U/L，白蛋白43 g/L，球蛋白38 g/L，碱性磷酸酶325 U/L；PET-CT示：躯干骨及所示四肢近段骨质密度不均匀，部分骨骼见虫蚀样骨质破坏，以右侧股骨头较明显，骨髓密度弥漫性增高，代谢活性不均匀轻度增高。给予唑来膦酸护骨及继续伊马替尼100 mg每日1次口服，骨痛逐渐减轻，现已恢复正常工作和生活半年余，门诊复查血常规和肝功能恢复正常，治疗6个月后于我院复查双髋关节平扫+增强磁共振结果示：双侧股骨干上段、髂骨、右侧髂骨翼多发异常信号，余病变较前好转。外周血FIP1L1::PDGFRα融合基因阳性（20％）。考虑到融合基因仍未转阴，建议患者将伊马替尼加量至200 mg每日1次治疗，患者拒绝，目前继续治疗随访中。

